# Therapeutic plasma exchange in a tertiary care center: 185 patients undergoing 912 treatments - a one-year retrospective analysis

**DOI:** 10.1186/s12882-017-0803-3

**Published:** 2018-01-15

**Authors:** Julius J. Schmidt, Firas Asper, Gunilla Einecke, Gabriele Eden, Carsten Hafer, Jan T. Kielstein

**Affiliations:** 1 Department of Nephrology and Hypertension, Medical School Hannover, Carl-Neuberg-Straße 1, 30625 Hannover, Germany; 2 Medical Clinic V | Nephrology | Rheumatology | Blood Purification, Academic Teaching Hospital Braunschweig, Braunschweig, Germany

**Keywords:** Immunological diseases, Plasma volume, Treatment cost

## Abstract

**Background:**

Therapeutic plasma exchange (TPE) is increasingly used throughout the world. Although the procedure itself is fairly standardized, it is yet unknown how the underlying disease entities influence the key coordinates of the treatment.

**Methods:**

Retrospective chart review. The treatment indications were clustered into four categories. Data are presented as median and interquartile (25–75%) range [IQR].

**Results:**

Within 1 year, 912 TPE treatments were performed in 185 patients (90 female, 48.6%). The distribution of the treatment numbers to the pre-specified disease categories were as follows: transplantation (35.7%), neurology (31.9%), vasculitis and immunological disease (17.3%), and others including thrombotic microangiopathy (8.1%), critical care related diseases (5.4%), hematology [multiple myeloma] (1.1%), and endocrine disorders (0.5%). The calculated plasma volume was significantly higher in patients with vasculitis and immunological diseases (3984 [3433–4439] ml) as compared to patients treated for transplant related indications (3194 [2545–3658] ml; *p* = 0.0003) and neurological diseases (3058 [2533–3359] ml; *p* < 0.0001). This was mainly due to the differences in the hematocrit which was 30.5 [27.0–33.6] % in the vasculitis/immunological disease patients and 40.2 [37.5–42.9] % in the neurological patients; *p* < 0.0001. Interestingly, treatment time using a membrane based technology was significantly longer than TPE using a centrifugal device 135.0 [125.0–140.0] min vs. 120.0 [112.5–135.0] min. Furthermore, the relative exchanged plasma volume was significantly lower in the treatment of vasculitis and immunological diseases as compared to treatments of transplant related indications and neurological diseases.

**Conclusion:**

Patients with low hematocrit and high body weight do not receive the minimum recommended dose of exchange volume. Centrifugal TPE allowed faster plasma exchange than membrane TPE.

## Background

Therapeutic plasma exchange (TPE) was first described as an extracorporeal blood purification technique more than a hundred years ago [1]. It removes pathogenic substances such as auto-antibodies, lipoproteins, and circulating immune complexes, from the plasma [2] and plays a key role in the management of various diseases. TPE can also replenish missing plasma components, e.g. ADAMTS13 in TTP, if fresh frozen plasma is used as exchange fluid. According to the 2016 guidelines of the American Society of Apheresis, it is the treatment of choice for acute ANCA associated rapid progressive glomerulonephritis, thrombotic thrombocytopenic purpura, Guillain-Barré syndrome, Goodpasture syndrome, and cardiac allograft rejection [3]. Treatment numbers are especially increasing in the transplant setting as the use of incompatible kidney transplantation is growing as a response to the organ shortage, hence more and more recipients of a live-donor kidney transplant as well as deceased donor organ need to be pretreated by TPE [4]. Periodically TPE treatment numbers are increasing in small epidemics such as the 2011 German STEC-HUS crisis. [5] Moreover, despite the lack of solid data, TPE is used in the intensive care setting for e.g. sepsis with multi organ failure or hypertriglyceridemia induced pancreatitis [6, 7] as it removes a plethora of pro-inflammatory cytokines [8].

As the previous guidelines, the 2016 ASFA recommendations suggest using TPE volume of 1.0–1.5 x the plasma volume of the patient [3]. There are only a few exceptions to that rule such as acute macular degeneration as well as the treatment of acute (mushroom) poisoning. To our knowledge, no studies have analyzed how these recommendations are followed in every day clinical practice, so far. Our assumption was that the underlying disease severity and perceived acuity may play a role in the determination of key coordinates of the TPE such as exchange volume in relation to the calculated plasma volume of the patient. Moreover, we were interested in whether the differences in treatment time between centrifugal TPE (cTPE) and membrane TPE (mTPE), recently shown in a prospective clinical study [9] could be confirmed under everyday conditions.

## Methods

### Study design

This is a retrospective analysis of all therapeutic plasma exchange (TPE) procedures at a tertiary care hospital performed between January 1st and December 31st 2012. One hundred eighty five patients with a total of 912 treatments have been evaluated. Written informed consent was waived by the Ethics committee of the Hannover Medical School due to the anonymized nature of the analysis.

Patients who received TPE were retrieved from the patient data management system of the hospital by entering the procedure code for TPE. For all identified patients the following information was entered into the studies data base:

Age (years), height (cm), weight (kg), Body-Mass-Index (kg/m^2^), laboratory markers such as hematocrit (%), the two modes to perform TPE (mTPE vs. cTPE), plasma exchange fluid (albumin vs. fresh frozen plasma or a mixture of both), the anticoagulants (citrate vs. heparin), the median treatment time (minutes), and the median of the exchanged plasma volume (ml).The following parameters were calculated: Plasma volume (PV): 0.065 x weight (in kg) x (1-hematocrit) and exchanged plasma volume pro time (ml/min).

### Statistical analysis

Data are presented as median and interquartile range [IQR]. Comparisons between both techniques were done by Wilcoxon and Mann-Whitney tests. The one-way analysis of variance (ANOVA) was used to determine statistically significant differences between the four treatment categories. Relationship between the exchanged and the calculated plasma volume was calculated by linear regression (r^2^) using GraphPad Prism (La Jolla, CA, USA), The significance levels was set at a *p*-values of <0.05.

## Results

**Fig. 1:**
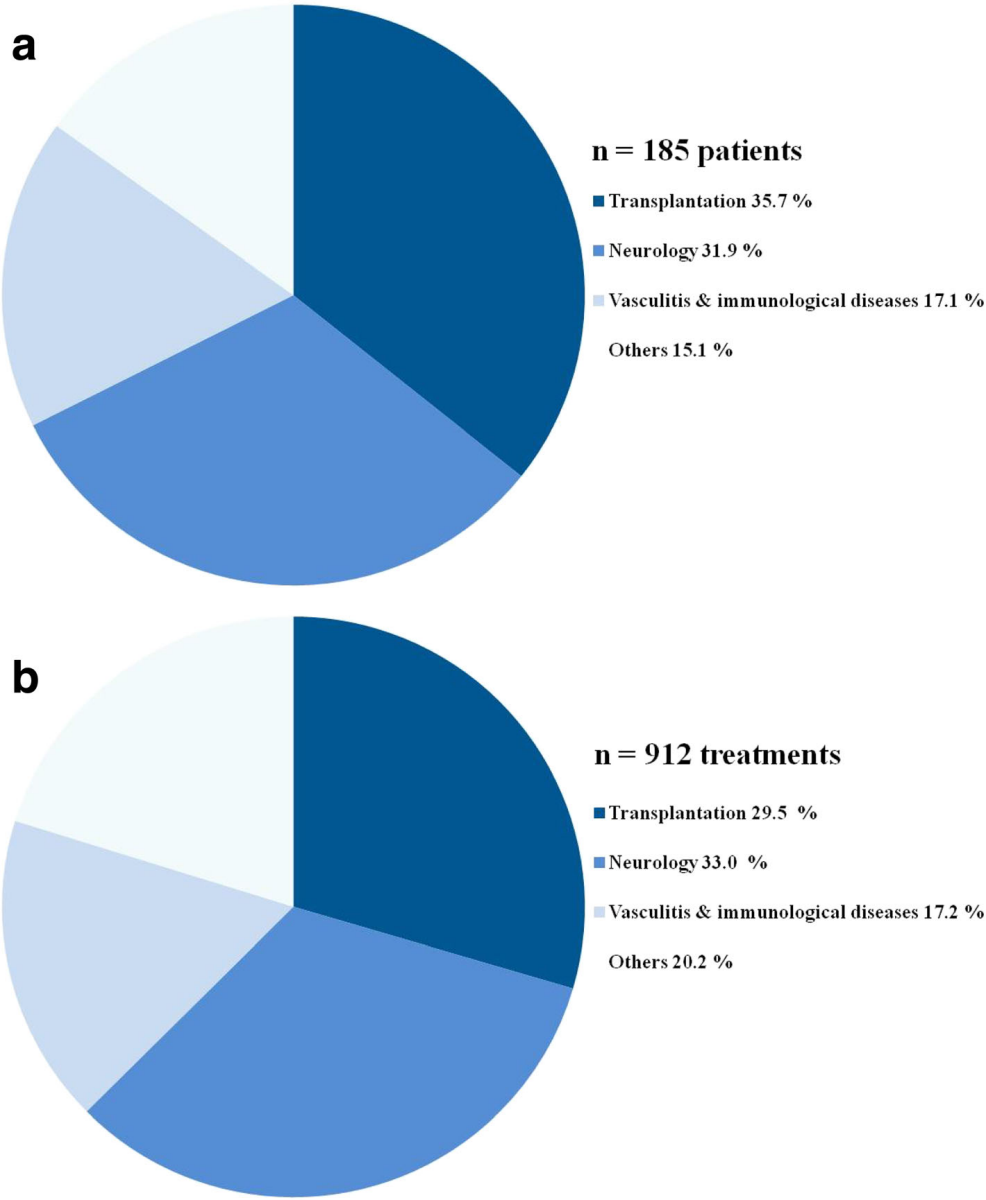
**a** Pie chart visualizing *the proportion patients* in the four disease categories. **b** Pie chart visualizing the *proportion of treatments* in the four disease categories

Within 1 year, 912 TPE treatments were performed in 185 patients (90 female, 48.6%). The spectrum of treated diseases was categorized into 4 groups. The percentage of patients in the pre-specified disease categories were as follows: transplantation (35.7%) [antibody mediated rejection, ABO incompatible transplantation], neurology (31.9%) [multiple sclerosis, acute disseminated encephalitis, Guillain-Barré syndrome], Vasculitis and immunological disease (17.3%) [ANCA- associated rapidly progressive glomerulonephritis, focal segmental glomerulosclerosis], and others including thrombotic microangiopathy (8.1%) [thrombotic thrombocytopenic purpura, atypical hemolytic uremic syndrome], critical care related diseases (5.4%) [sepsis with multiorgan failure, ARDS], hematology [multiple myeloma] (1.1%) and endocrine diseases [thyroid storm] (0.5%) (Fig. 1a).

In accordance with the frequency of the four different disease categories the number of treatments was highest in the neurology group (33.0%), followed by the transplantation group (29.5%) and others (20.3%), as well as vasculitis and the immunological group (17.2%) (Fig. 1b).

### Calculated and exchanged plasma volume

**Fig. 2:**
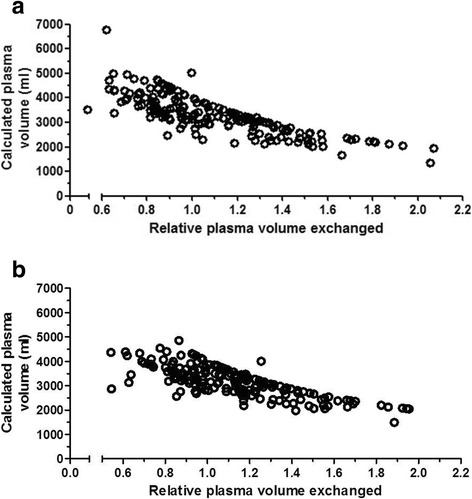
**a** Relationship between the relative plasma volume exchanged and absolute calculated plasma volume (based on the body weight and hematocrit) (r^2^ 0.6438, *p* < 0.0001. 45% of treatments were done with an exchange volume < 1.0 times the calculated plasma volume. **b** Relationship between the relative plasma volume exchanged and absolute calculated plasma volume (based on the Nadler formula) (r^2^ 0.5940, *p* < 0.0001. 37% of treatments were done with an exchange volume < 1.0 times the calculated plasma volume

The patients’ calculated plasma volume (Table 1) was significantly higher in patients with vasculitis and immunological diseases (3984 ml [3433–4439]) as compared to patients treated for transplant related indications (3194 ml [2545–3658]; *p* = 0.0003) and neurological diseases (3058 ml [2533–3359]; *p* < 0.0001). This was mainly due to the differences in the hematocrit which was 30.5% [27.0–33.6] in the vasculitis/immunological disease patients and 40.2% [37.5–42.9] in the neurological patients; *p* < 0.0001. Thus, the relative exchanged plasma volume (Table 1) was significantly lower in patients with vasculitis and immunological diseases (0.87 [0.79–0.99]) as compared to patients treated for transplant related indications (1.16 [0.94–1.34]; *p* < 0.0001) and neurological diseases (1.17 [0.97–1.38]; *p* < 0.0001). In general, there was an inverse relationship between the actual (relative) plasma volume exchanged and the calculated plasma volume (Fig. 2). Interestingly, while the calculated plasma volume using the NADLER formula was 3131 [2708–3594] ml it was significantly higher when the standard formula was used 3240 [2737–3849] ml (*p* value <0.0001). Further the relationship between the relative plasma volume exchanged and absolute calculated plasma volume also differed depending on the underlying formula used. It was r^2^ 0.6438, *p* < 0.0001 (Fig. 2a), meaning that 45% of treatments were done with an exchange volume < 1.0 times the calculated plasma volume. In contrast, when using the NADLER formula was used, the linear regression was r^2^ of 0.5940, *p* < 0.0001 (Fig. 2b). Using this formula 37% of treatments were done with an exchange volume < 1.0 times the calculated plasma volume.

**Table 1:** Characteristics of the study population. All values are given as median [IQR] unless otherwise stated

	All (*n* = 185)	Neurology (*n* = 59)	Transplantation (*n* = 66)	Vasculitis and Immunological Disease (*n* = 32)	Others (*n* = 28)	One way ANOVA *P* value
Age (years)	54.0 [43.0–64.0]	56.0 [39.0–67.0]	54.0 [47.0–62.0]	54.5 [47.3–64.5]	51.5 [32.5–63.8]	0.2738
Sex (m/f)	95/90	29/30	35/31	16/16	15/13	NS
BMI (kg/m^2^)	25.5 [22.4–29.3]	24.8 [21.7–28.2]	24.3 [22.3–27.8]	26.1 [22.6–30.6]	24.7 [22.0–28.4]	0.1416
Hct (%)	33.9 [29.8–39.2]	40.2 [37.5–42.9]	33.7 [31.0–37.0]	30.5 [27.0–33.6]	24.6 [20.2–31.2]	< 0.0001
PV_calc_ (ml)	3240 [2737–3849]	3058 [2533–3359]	3194 [2545–3658]	3984 [3433–4439]	3699.5 [3125–4149]	< 0.0001
PV_calc_ NADLER (ml)	3131 [2708–3594]	2846 [2542–3182]	3083 [2543–3571]	3501 [3199–3972]	3631 [3123–3916]	< 0.0001
PV_ex_ (ml)	3500 [3028–3987]	3599 [3101–4000]	3600 [3165–4000]	3443 [3037–3893]	2998 [2823–3576]	< 0.0001
PV_ex_/PV_calc_	1.01 [0.86–1.28]	1.17 [0.97–1.38]	1.16 [0.94–1.34]	0.88 [0.79–0.99]	0.83 [0.70–0.96]	< 0.0001
PV_40ml/kg_	3080 [2600–3560]	3080 [2580–3420]	2950 [2465–3285]	3540 [3140–3990]	3174 [2630–3480]	0.0004
PV_calc_/PV_40ml/kg_ (%)	+ 4.94%	- 0.72%	+ 7.64	+ 11.2%	+ 14.21%	*p* <0.0001
PV_ex_/PV_40ml/kg_ (%)	+ 12.00%	+ 14.42%	+ 18.06%	- 2.82%	- 5.91%	*p* <0.0001
Treatments (n)	5 [3–5]	5 [5–5]	4 [3–5]	5 [3–7]	4 [2–8]	0.0078
1st - 5th TPE (d) (*n= 61*)	7 [6–10]	7.5 [7–10]	7 [6–11]	6.5 [6–7.75]	4 [4–5.5]	*p* <0.0001
Exchange fluid (%)						
Albumin	54.1	88.4	56.2	37.6	9.2	*p* <0.0001
FFP	34.7	7.6	22.1	45.2	87.0	*p* <0.0001
Mix	11.2	4.0	21.7	17.2	2.7	*p* <0-0001

### Treatment time and number of treatments

**Fig. 3:**
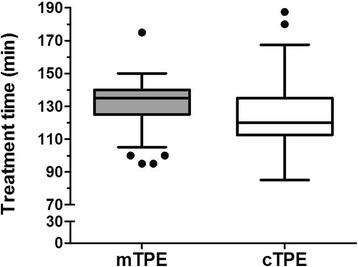
Median [IQR] treatment time in patients receiving membrane base TPE (mTPE) and centrifugal TPE (cTPE); difference by two tailed *t*-test *p* = 0.007

Interestingly, treatment time using a membrane based technology (mTPE) was significantly longer than TPE using a centrifugal device (cTPE) 135.0 [125.0–140.0] min vs. 120.0 [112.5–135.0] min; *p* = 0.007 (Fig. 3). The time between the first and the last treatment was longer in neurological patients 7.5 [7–10] d as compared to patients receiving TPE for the disease group of others related causes 4 [4–5.5] d (Table 1).

### Adverse events

Adverse events occurred in 16.22% of the patients (4.39% of all the treatments). The majority of the 40 adverse events occurred in the transplantation group. Severe adverse events leading to the discontinuation of the treatment occurred in two of the 185 patients (Table 2). The mode of TPE, i.e. mTPE vs. cTPE, seems to influence the adverse event rate. While 1.91% of the cTPE treated patients had an adverse event during the procedure, this rate was significantly higher in the mTPE group (5.22%). Adverse event rate was not affected by the type of exchange fluid (albumin: 2.19%; fresh frozen plasma: 1.09%; mix of albumin and fresh frozen plasma: 1.09%). As all patients treated with cTPE received anticoagulation by citrate, a differentiation between the method of treatment (cTPE vs. mTPE) and the anticoagulant (heparin vs. citrate) could not be calculated.

**Table 2:** Adverse events and their severity. We divided the adverse events in 34 *mild and moderate* (transient but clearly visible, considerable discomfort and requirement of medication, no termination of procedure required) and 6 *severe* (clinically unstable due to adverse event OR termination of procedure required)

Adverse events	Severe adverse events	Mild and moderate adverse events
Abdominal Pain	0	1
Allergic reactions	1	2
Angina pectoris	1	0
Convulsions	1	0
Dysesthesia	0	7
Dyspnea	1	1
Fever	1	1
Headache	0	2
Hypotension	1	2
Hypocalcemia	0	2
Nausea/Vertigo	0	4
Others	0	12

## Discussion

The pertinent findings of our analysis were as follows: i) plasma volume and exchanged volume during TPE differ depending on the underlying disease category, ii) cTPE treatment time was lower than mTPE treatment time, and iii) complications were rare and partially dependent on the treatment modality and the underlying disease being treated iv) a low hematocrit and a high bodyweight were factors associated with not delivering at least 1.0 times of the calculated plasma volume,

With 912 TPE performed in 185 patients we here present one of the largest datasets on TPE. Thus far, the largest four studies were by Arslan et al. who reported on 658 TPEs in 158 patients over a 3 year period, [10] followed by a study from Colombia reporting 500 TPE sessions in 68 patients over a period of 5 years, [11] a study from India reporting in 492 TPE procedures performed in 125 patients, [12] and the study by Stegmayr et al. who reported on 388 TPE in 122 patients [13].

### Plasma exchange volume

Data on exchanged plasma volume during TPE are rare. One of the few available studies is an open prospective observational study from 1987 to 1989 in East Germany. With 1945 procedures in a total of 419 patients (on average 4.1 treatments per patient) about 80% of all treatments in the country were registered. Exchange volume averaged 2.7 (+/− 0.78) l or 43 (+/− 13.9) ml/kg body weight. Substitutes were albumin (51% of the cases), FFP (22%), and both (22%) [14].

Our analysis shows that neither (low) hematocrit, nor (high) body weight are fully taken into account when prescribing the individual exchange volume. Most of the patients receive an exchange volume that is within the ASFA recommended range. However, especially patients with severe underlying diseases such as vasculitis and immunological disease, receive less than the suggested dose of at least 1.0 times the calculated plasma volume. This is especially disturbing as an ongoing randomized controlled study evaluating the role of TPE in patients with ANCA-vasculitis suggests exchanging the plasma volume based on body weight only (60 ml/kg) [15]. If this approach would have been applied to the vasculitis patients in our cohort the exchange volume should have been 5340 [4680–6000] ml were in fact we exchanged only 3500 [3028–3987] ml, i.e. 66% of the recommended exchange volume in the PEXIVAS study. It is of little comfort that the PEXIVAS recommended exchange volume would correspond to this would represent 1.65 times of the calculated plasma volume (3240 [2737–3849] ml), i.e. being even higher than the upper limit of the current ASFA recommendations. Hence an individually calculated exchange volume based on actual body weight and hematocrit seems to be a more prudent approach than a calculation of plasma volume based on body weight only. Aside from the failure to calculate individually plasma volume based on both, body weight and hematocrit there are other factors that might have an impact. Economic restraints, i.e. a fixed reimbursement of the procedure, independent on the amount, i.e. cost of blood products may lead to rather fixed exchange volumes in daily practice [16]. Even tertiary care centers admit to exchange only 0.4–1.0 times the calculated plasma volume of their patients [17]. A recent study from India reports an overall exchange volume as low as 2097 ± 916 ml [12]. Although, the calculated plasma volume is not reported by the authors, the average patient in this population must have a body weight of 40 kg with a low hematocrit of 35% to let this be a sufficient TPE dose.

### Treatment time and number of treatments

The required treatment time of TPE has rarely been investigated. Given today’s growing economic pressure, it is yet an increasingly relevant topic. In two small randomized prospective trials, a significantly lower required treatment time and a consecutively higher exchanged plasma volume per time was described for cTPE compared to mTPE treatments [9, 18]. Even though the median 15 min difference in treatment time we found in our analysis is almost irrelevant if a hospital performs only a few treatments per year, this difference can become relevant if dozens or hundreds of treatments are performed, given the high costs that for skilled labor. Interestingly, there was also a difference in treatment duration looking at the whole treatment cycle, which is the longest in neurological patients. One underlying reason was the type of replacement fluid used, which was albumin in 88.4% of all neurological patients. This frequently necessitates pausing TPE to wait for fibrinogen levels to come back to minimum threshold levels preventing bleeding complications.

### Treatment related adverse events

Compared to the published literature, the reported rate of side effect rate in our study is rather low (4.39% of all treatments). A higher adverse event rate during the treatment with fresh frozen plasma as published in many studies could not be confirmed I our cohort as over 50% of all side effects occurred during therapy with heparin anticoagulation. Possible reasons may be the liberal usage of constant calcium infusions during the treatment with citrate anticoagulation and the administration of prednisolone prior to the substitution of fresh frozen plasma during TPE in our hospital. The percentage of treatment related adverse events described in the literature ranges dramatically, depending on the clinical setting, the era the study was performed, and the definition of side effects. A complication rate of 11% is reported in the world apheresis registry report of 2007, [13] while more than a third of all treatments in a Chinese cohort have allegedly side effects (36%) [19]. However, only 1.6% of the events, reported in this study were severe, therefore, it is important to distinguish between rather mild side effects such as hypocalcemia with tingling sensations or moderate adverse events in patients that need calcium supplementation. Interestingly, if adverse events due to lower calcium were removed from the apheresis registry report, only 6.4% adverse events occurred.

### Limitations of the study

We wish to point out important limitations of our study. Firstly, this study is retrospective in nature. Further, as we have not interviewed individual prescribers of TPE we could no elucidate the causes/reasoning that leads to a prescription of a lower exchange volume in patients with vasculitis and immunological diseases. Lastly, as this was not an economical study we cannot provide a detailed cost analysis of how the variation of the exchange volume would influence the actual cost of the TPE.

## Conclusions

The exchange plasma volume should be individually calculated for all patients bearing in mind the effect of different formulas as well as key variables such as weight and hematocrit. In patients with low hematocrit calculating the exchange volume on body weight only will results in the delivery an inadequate, i.e. low exchange volume. About one third of patients receives less than the currently recommended lowe limit of the exchange volume (1.0.x calculated plasma volume). For units performing many TPEs per year, the shorter treatment time of cTPE as compared to mTPE could have an economical impact by saving nursing time.

## Abbreviations

Alb: Albumin
ARDS: Acute respiratory distress syndrome
ASFA: American Society for Apheresis
BCT: Blood compotent and cellular technologies
BMI: Body Mass Index
cTPE: Centrifugal therapeutic plasma volume
Ex fluid: Exchange fluid used
FFP: Fresh frozen plasma
Hct: Hematocrit
IQR: Interquartile range
Mix: Mixture of albumin and fresh frozen plasma
mTPE: Membrane based therapeutic plasma exchange
PV: Plasma volume
PV_40ml/kg_: Calculated exchange volume for 40 ml/kg/body weight
PV_calc_: Calculated plasma volume
PV_ex_: Exchanged plasma volume
STEC: Shiga toxin producing *Escherichia coli*
TPE: Therapeutic plasma exchange
TTP: Thrombotic thrombocytopenic purpura
